# The Effects of Grain Size and Twins Density on High Temperature Oxidation Behavior of Nickel-Based Superalloy GH738

**DOI:** 10.3390/ma13184166

**Published:** 2020-09-19

**Authors:** Wenbin Ma, Hongyun Luo, Xiaoguang Yang

**Affiliations:** 1 School of Energy and Power Engineering, Beihang University, Beijing 100191, China; binwenma2008@126.com (W.M.); yxg@buaa.edu.cn (X.Y.); 2 School of Materials Science and Engineering, Beihang University, Beijing 100191, China; 3 Beijing Advanced Innovation Centre for Biomedical Engineering, Beihang University, Beijing 100191, China

**Keywords:** GH738 superalloy, low temperature burnishing, nano-grains, nano-twins, high temperature oxidation

## Abstract

In the present study, surface treatment techniques such as room temperature machining (RTM) and low temperature burnishing (LTB) processing have been used to improve the microstructure of GH738 superalloy. Nano-grains and nano-twins are obtained on the top surface of RTM and LTB specimens. It is found that although the grain size of RTM and LTB specimens is almost the same, different types of nano-twins have been produced. Moreover, the effect of RTM and LTB processing on high temperature oxidation behavior of nickel-based superalloy GH738 at 700 °C is investigated. The result shows that LTB specimen has the best high temperature oxidation resistance owing to the formation of nano-grains and higher twins density, which induce to form a continuous protective Al_2_O_3_ layer at the interface between outer oxide layer and matrix. It is observed that this layer inhibits the inward diffusion of O and outward diffusion of Ti and significantly improves oxidation resistance of LTB specimen. Furthermore, the effects of nano-grains and crystal defects on the diffusion mechanism of elements are clarified during the high temperature oxidation test.

## 1. Introduction

Nickel-based superalloys (GH738 (Chinese code)) are extensively used as the blades and disks of industrial jet engines and gas turbines due to their excellent properties such as fatigue properties [1,2], hot corrosion resistance [3,4,5,6,7], and oxidation resistance at elevated temperatures [8,9,10,11]. However, these alloys degrade during the long-term service in harsh environments. The main failure modes are fatigue fracture, high temperature oxidation, and hot corrosion. Further investigation shows that these failures, which significantly limit the performance and service life of alloys, are closely related to the surface microstructure (grain size and crystal defects) [12]. Therefore, many surface treatment techniques (STTs) [13] such as laser shock peening (LSP) [14,15], surface mechanical attrition treatment (SMAT) [16,17,18], surface mechanical rolling treatment (SMRT) [19], surface mechanical grinding treatment (SMGT) [20,21], ultrasonic shot peening (USSP) [22], machining [23], low temperature (cryogenic temperature) burnishing (LTB) [24,25] and shot peening [26] have been used to change the surface microstructure of alloys by means of severe plastic deformation [27].

In the past few years, coarse grains have been refined to multiscale grains, ranging from several nanometers to micrometers in the surface layer of alloys by these STTs. It can significantly affect the fatigue properties [22,28], high temperature oxidation resistance [29,30], and hot corrosion behavior [31] of alloys. As for fatigue life, it has been reported by Ren et al. [32] and Zhou et al. [28] that the fatigue life of steel [32] and GH4133B [28] after LSP is higher than that of untreated samples due to the surface grain refinement and more stable dislocation arrangement. Moreover, Ritchie et al. [33] demonstrated that the burnishing processing significantly improves the fatigue life of Ti-6Al-4V, even at temperatures as high as 550 °C. Nevertheless, there are two disputed results about the influence of different STTs on high temperature oxidation [34,35,36]. Hua et al. [34] and Tan et al. [35] investigated that the high temperature oxidation resistance of GH586 whose average grain size is 18.5 μm after LSP treatment and alloy 800H whose average grain size is 20 nm after shot peening has been improved, the reason of which could be the selective oxidation of Cr to form protective Cr_2_O_3_. In contrast, Wu et al. [36] announced that the oxidation process of K38G at 1000 °C has been accelerated after the sand blasting. The above studies show that the change in grain size may have different influences on the high temperature oxidation resistance of the alloys. It is of great significance to further study the effect of grain refinement induced by different surface treatment techniques on high temperature oxidation behavior of alloys.

To compare with room temperature burnishing, liquid nitrogen is applied at the contact interface between the tool and the sample surface to provide a cryogenic environment during the burnishing process [25,37]. This process is defined as low temperature burnishing (LTB). The processing of LTB which can induce a nanocrystalline layer on the surface of alloys has been developed as a rapid, chipless, and inexpensive STT [37,38]. Recent studies have focused on applying LTB to improve the surface integrity and corrosion resistance of alloys. Jawahir et al. [37] reported that refined grain structure and an improved surface finish are achieved in the severe plastic deformation layer produced by LTB. Pu et al. investigated [39] that an ultrafine-grained surface layer is produced on Mg-Al-Zn alloy by LTB and the corrosion resistance is significantly enhanced. Ritchie et al. [40] found that the nanocrystalline structure formed by LTB could maintain thermal stability, even at high temperatures. Our previous work [38,41,42] also showed that the LTB could improve the surface integrity, such as roughness, hardness, and corrosion resistance of alloys. In addition, one surface treatment technique (RTM) can be used to modify the surface microstructure of alloys. Swaminathan et al. [23] demonstrated that the surface grains of Inconel 718 have been refined and hardness has been improved by RTM. Nouduru et al. [43] reported that fine-grained structure which results in oxidation resistance of Zr-2.5Nb alloy improved has been produced by RTM. However, the high temperature oxidation of GH738 treated by LTB has been scantily studied thus far. 

In this work, it is intended to treat GH738 superalloy by LTB and RTM and then study the surface microstructure of treated specimens. High temperature oxidation behavior of GH738 treated by LTB and RTM and untreated is investigated in static air at 700 °C. It is expected to investigate the effect of nano-grains and crystal defects (such as twins density) on the elements diffusion during the high temperature oxidation test, which may provide new insights into improving oxidation resistance at elevated temperatures.

## 2. Experimental Procedure

### 2.1. Materials

**Figure 1:**
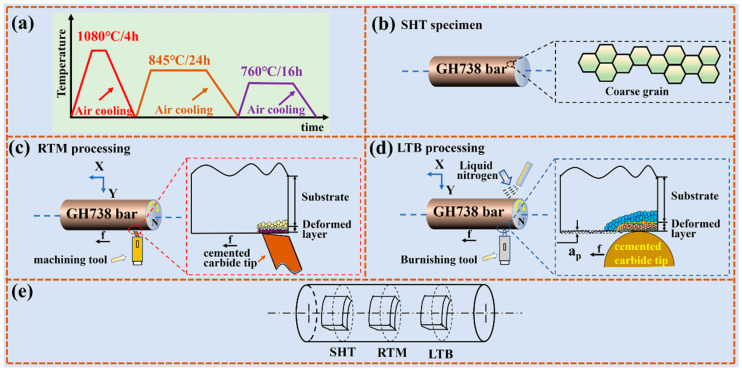
The schematic of high temperature oxidation: (**a**) solution heat treatment process; (**b**) solution heat treated specimen; (**c**) room temperature machining processing; (**d**) low temperature burnishing processing; (**e**) specimen cutting.

GH738 superalloy used in this work is in bar form with a diameter of 50 mm. The chemical composition is given in Table 1. The prepared bars initially undergo solution heat treatment in three steps. It is firstly kept at 1080 °C for 4 h, followed by holding at 845 °C for 24 h, and finally at 760 °C for 16 h. Each step is followed by an air cooling process. The schematic of heat treatment process is shown in Figure 1a. The solution heat treated specimen is named as SHT specimen (Figure 1b).

### 2.2. Surface Treatment Process

In this work, the processing of RTM and LTB are employed to obtain the nano-crystallization layer on the GH738 superalloy surface with a cemented carbide tool. In order to provide standard initial machining and burnishing processing conditions, the topmost surface of GH738 superalloy bar is skimmed off before RTM and LTB processing (about 2 mm). The schematic of RTM and LTB processing are given in Figure 1c,d, respectively. The specimen rotates around the *x*-axis with a rotating velocity of *N* and then the cemented carbide tool slowly slides along the positive *x*-axis with a speed of *f*. The carbide tip penetrates into the specimen at an appropriate preset depth *a_p_*, resulting in the formation of the plastic deformation zone under the tip. The parameters of RTM are set as follows: *N* = 400 rev/min, *f* = 40 μm/rev, and *a_p_* = 20 μm. The parameters of LTB processing are set as: *N* = 400 rev/min, *f* = 5 μm/rev, and *a_p_* = 20 μm. It should be indicated that the burnishing processing is carried out at a low temperature (liquid nitrogen, LT) to obtain a nanocrystal microstructure. The detailed burnishing processing could be referred to our previous papers [38,44,45]. The specimens obtained by RTM and LTB processing are hereafter named as RTM and LTB specimens, respectively. Then, the electric discharge wire cutting is used to cut the SHT, RTM, and LTB specimens from the surface of the superalloy bar. The specimens cutting is schematically presented in Figure 1e.

### 2.3. Oxidation Test

The oxidation experiments are carried out at 700 °C in static air up to 100 h in a silicon carbide furnace. Three types of specimens (SHT, RTM, and LTB specimens) are used in this study. Before oxidation tests, specimens are washed with alcohol and are ultrasonically cleaned in acetone. The alumina crucibles are preheated for 24 h at 750 °C to remove any possible moisture. Therefore, the mass of the crucibles is assumed constant during the experiments. The specimens are weighted at 2 h, 4 h, 6 h, 8 h, 10 h, 25 h, 50 h, 75 h, and 100 h, using an electronic balance with an accuracy of 0.01 mg after cooling down to room temperature by 30 min. The mass change per unit area of three parallel specimens is averaged for each type.

### 2.4. Microstructure Characterization

After oxidation tests, the surface and cross-sectional microstructure and elements distribution of GH738 are examined under a field emission scanning electron microscope (SEM, Zeiss supra55, Zeiss Corporation, Jena, Germany) equipped with energy dispersive X-ray spectroscope (EDS, Oxford Instruments Corporation, Oxford, UK). Each specimen is cold mounted in epoxy resin, ground up to 2000 grit, and polished with 1.5 mm diamond paste. The grain size and twins morphology are investigated using transmission electron microscopy (TEM, JEM-2100F, Japan Electronics Corporation, Tokyo, Japan) at 200 kV acceleration voltage. The X-ray diffraction (XRD) is used to identify the oxidation product formed on the surface of the corroded specimens after oxidation at 700 °C. The microstructure of the surface layer of SHT, RTM, and LTB specimens are also investigated by XRD. The XRD patterns are obtained using a Rigaku D/max-2500 diffractometer (Rigaku Corporation, Tokyo, Japan) with Cu Kα radiation at 40 kV and 200 mA. The scanning speed is 6°/min. 2θ is scanned from 20° to 80°.

## 3. Results and Discussion

### 3.1. Surface Layer Characterization

**Figure 2:**
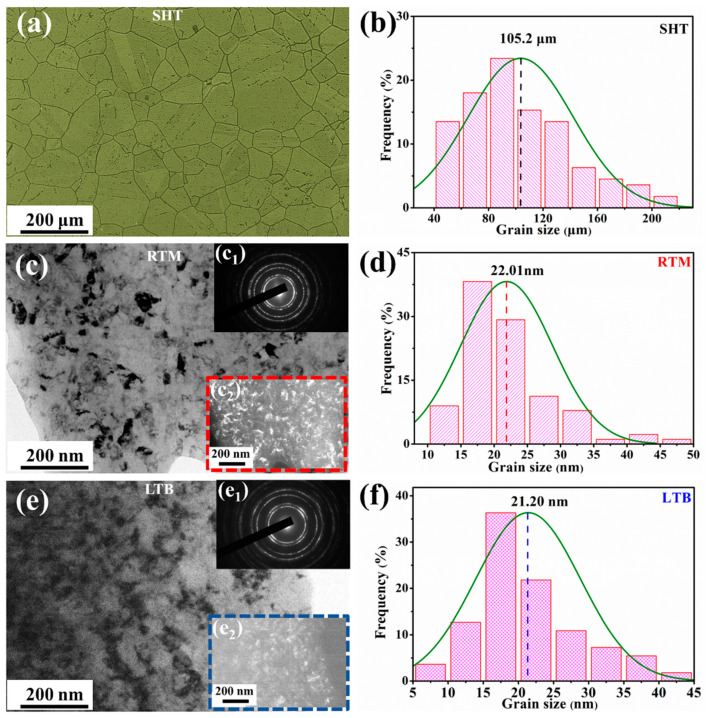
Optical Microscopy observations of the solution heat treated (SHT) specimen and TEM observations of the room temperature machining (RTM) and low temperature burnishing (LTB) specimen surfaces: (**a**,**b**) OM image and the grain size distribution of the topmost layer on the SHT specimen; (**c**,**d**) bright-field TEM image and the grain size distribution of the topmost layer on the RTM specimen; (**c_1_,c_2_**) the selected area electron diffraction pattern and dark-field TEM image of the topmost layer on the RTM specimen; (**e**,**f**) bright-field TEM image and the grain size distribution of the topmost layer on the LTB specimen; (**e_1_**,**e_2_**) the selected area electron diffraction pattern and dark-field TEM image of the topmost layer on the LTB specimen.

The microstructure and the grain size distribution of these three types of GH738 specimens are shown in Figure 2. After solution heat treatment, the average grain size of the SHT specimen is about 105.2 μm (Figure 2a,b). TEM is carried out on the top surface of the RTM and LTB specimens to further characterize their microstructure as shown in Figure 2c,e. Figure 2c–f illustrate microstructure and grain size distribution of the RTM and LTB specimens, respectively. The grain sizes of the RTM and LTB specimens are obtained by statistical measurement of the bright areas in the dark-field images (Figure 2c_2_,e_2_) for more than 200 grains. As can be seen from Figure 2c_1_,e_1_, the homogeneous, continuous, and broadened concentric rings in the selected area electron diffraction (SAED) pattern indicate that the grain has been refined [46] due to the severe plastic deformation induced by RTM and LTB processing in the surface layer. It should be noted that uniform nano-grains appear in the surface layer after RTM and LTB processing. The statistical results show that the average grain sizes of the RTM and LTB specimens are 22.01 nm and 21.20 nm, respectively (Figure 2d,f).

**Figure 3:**
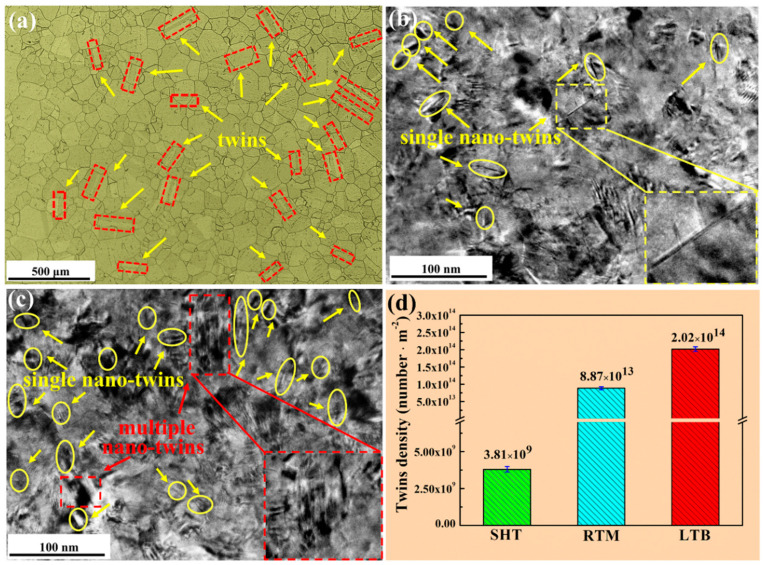
The different scales of twins in (**a**) the SHT specimen; (**b**) the RTM specimen; and (**c**) the LTB specimen and (**d**) the twins density.

Different scales of twins in SHT, RTM, and LTB specimens are displayed in Figure 3. According to the research of Meng et al. [47], the number of twins per unit area can be used to describe twins density of the SHT, RTM, and LTB specimens. There are many micro-scale annealing twins in the SHT specimen shown in Figure 3a, with twins density of 3.81 × 10^9^ number/m^2^. Nano-twins are twins whose length and thickness are at the nanoscale [19,48]. Typical nano-twins could be found in specimens subjected to RTM and LTB processing in Figure 3b,c. It can be seen from Figure 3b that the RTM specimen forms single nano-twins (marked by yellow arrows), with twins density of 8.87 × 10^13^ number/m^2^. As shown in Figure 3c, the LTB specimen forms single nano-twins (marked by yellow arrows) and multiple nano-twins (marked by red dash), and the twins density is 2.02 × 10^14^ number/m^2^.

It should be indicated that the formation of nano-twins is derived from ultrahigh strain rate and high peak pressure induced by RTM and LTB processing. The ultrahigh strain rate suppresses the dislocation slip and high peak pressure provides high enough driving energy for twinning [49].

### 3.2. Oxidation Kinetics Analysis

**Figure 4:**
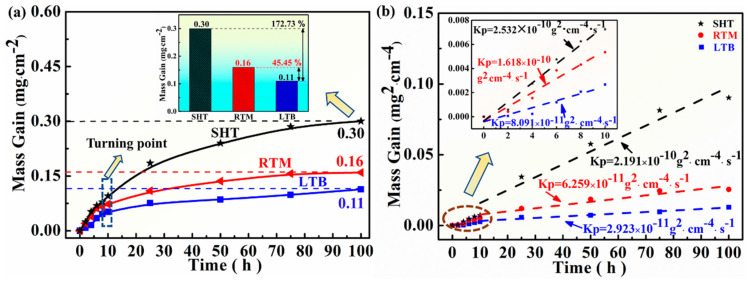
The oxidation kinetic curves of SHT, RTM, and LTB specimens at 700 °C. (**a**) Mass gain versus oxidation time; (**b**) mass gain square versus oxidation time.

The oxidation kinetics of the SHT, RTM, and LTB specimens at 700 °C is illustrated in Figure 4. The oxidation kinetics are determined through the relationship of mass gain versus oxidation time. As shown in Figure 4a, the mass gain increases whereas mass gain rate decreases as time extends. The mass gain value of SHT is significantly higher than that of the other two types. After oxidation for 100 h at 700 °C, the average mass gain value of the SHT specimen is 0.3 (mg/cm^2^), 45.45% higher than that of RTM specimen, and 172.73% higher than that of the LTB specimen. It indicates that the SHT specimen has the worst oxidation resistance among the three kinds of specimens. It is observed from Figure 4b that the oxidation kinetics of GH738 nearly follows parabolic law, indicating that the oxidation process is mainly determined by the diffusion [50]. The oxidation rate can be calculated by Equation (1) [4]:

(Δ*m*/*A*)^2^ = *K_p_*∙*t* + *C* (1)

where (Δ*m*/*A*) is mass gain per unit area; *K_p_* is parabolic rate constant; and *C* is a constant. (Δ*m*/*A*) is measured in mg∙cm^−2^ and time *t* in seconds. The *K_p_* is calculated in g^2^cm^−4^s^−1^. 

Furthermore, Figure 4a shows that the mass gains of the three types of specimens exhibit a relatively rapid increase in the initial 10 h. In addition, the SHT specimen has the largest mass gain while the LTB specimen has the smallest one. In the initial 10 h, the *K_p_* of SHT, RTM, and LTB specimens are determined to be 2.532 × 10^−^^10^, 1.618 × 10^−^^10^, and 8.091 × 10^−^^11^ g^2^cm^−^^4^s^−^^1^, respectively (Figure 4b). Then, further mass gain of these specimens is relatively low which means that the oxidation process transits into to a steady stage, with *K_p_* of 2.191 × 10^−^^10^, 6.259 × 10^−^^11^, and 2.923 × 10^−^^11^ g^2^cm^−^^4^s^−^^1^, respectively (Figure 4b). In steady stage, a continuous stable protective oxide layer is formed [51]. The lower the value of *K_p_*, the higher the oxidation resistance and vice versa [4]. The *K_p_* value of LTB specimen is found to be lower than that of RTM and SHT specimens, which indicates that the LTB specimen has the best oxidation resistance compared to the other specimens. 

### 3.3. Phase Constitution of Surface Oxidation Product

**Figure 5:**
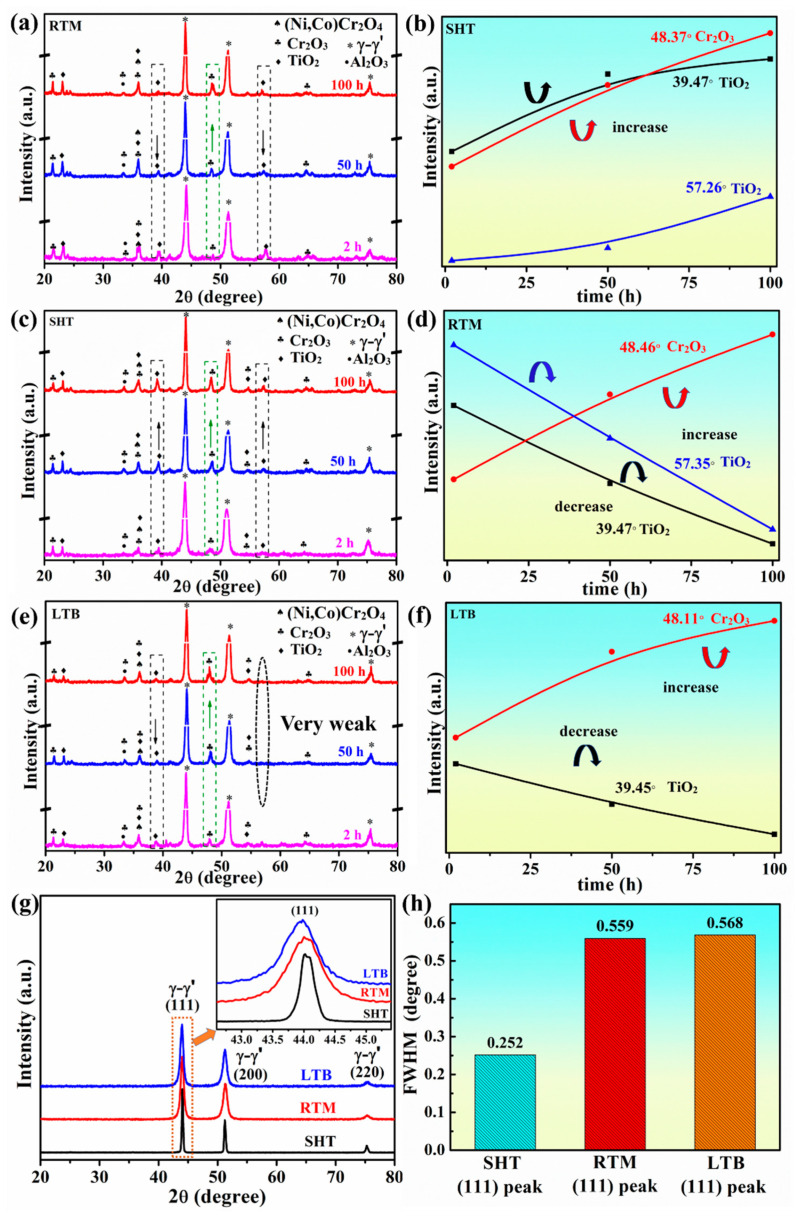
(**a**–**f**) XRD patterns of oxidation product; (**g**) XRD patterns of GH738 specimens under different surface treated and (**h**) FWHM values of (111) peak of SHT, RTM, and LTB specimens.

High temperature oxidation resistance of superalloys is primarily determined by the protective oxide scale formed on the surface of the superalloys [52]. Moreover, the phase composition of the oxidation product is confirmed by XRD. Figure 5 illustrates the XRD results for the surface oxidation product of all three types of specimens after oxidation test at 700 °C.

The XRD patterns (Figure 5a,c,e) show that after the oxidation test, the oxidation product on the surface of SHT, RTM, and LTB specimens consist of Cr_2_O_3_, TiO_2_, (Ni,Co)Cr_2_O_4_, and Al_2_O_3_. Al_2_O_3_ and Cr_2_O_3_ [10,53], which are considered to be the protective oxide for their stable chemical properties at elevated temperatures, inhibit further oxidation of the matrix. Compared with Al_2_O_3_ and Cr_2_O_3_, TiO_2_ is considered to be the unprotective oxide. TiO_2_ loosens the outer oxide layer and provides the path for oxygen to diffuse into matrix [10,54]. It should be indicated that (Ni,Co)Cr_2_O_4_ spinel is formed through the solid phase reaction between (Ni,Co)O and Cr_2_O_3_ [50]. At high temperatures, Co and Ni have similar physical properties, which result in similar diffusion behaviors. Therefore, they coexist in the oxidation product to form (Ni,Co)Cr_2_O_4_ [6]. 

The XRD curve is normalized, and the relative peak intensity can be used to represent the relative content of oxide [34]. In Figure 5, the black arrow and the green arrow represent the variation of the characteristic diffraction peaks of TiO_2_ and Cr_2_O_3_, respectively. Figure 5a,b shows that the content of TiO_2_ and Cr_2_O_3_ on the surface of SHT specimen gradually increases during the experiment. However, Figure 5c–f indicates that the content of Cr_2_O_3_ on the surface of RTM and LTB specimens gradually increase as the time extends, while the content of TiO_2_ gradually decreases.

The analysis of FWHM (full width at half maximum) can be used to investigate the effect of surface treatment techniques on the grain size change [26,55]. As shown in Figure 5g, the matrix (111), (200), and (220) peaks become broad after RTM and LTB treatment. The magnified view in Figure 5g shows the peak broadening difference at the matrix (111) peak. The FWHM values of matrix (111) peak of SHT, RTM, and LTB specimens are shown in Figure 5h. According to the FWHM values (Figure 5h) and Scherrer’s equation [56], it can be inferred that the grains have been refined after RTM and LTB treatment.

### 3.4. Surface Morphology and Composition

**Figure 6:**
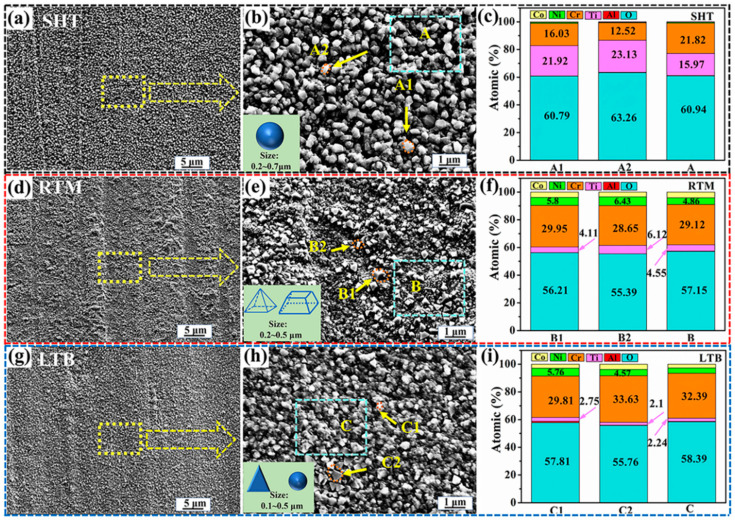
Surface morphology after high temperature oxidation of 100 h and results of EDS: (**a**–**c**) SHT specimen; (**d**–**f**) RTM specimen; and (**g**–**i**) LTB specimen.

Figure 6a,d,g show the surface morphology of SHT, RTM, and LTB specimens, respectively, after oxidation at 700 °C for 100 h. Figure 6b,e, and h are the magnified image of the dotted frame zone in Figure 6a,d,g, respectively.

It can be seen from Figure 6b that lots of spherical oxides loosely distribute on the surface of SHT specimen, and the sizes of oxides range from 0.2 to 0.7 μm. The EDS results from Figure 6c (Point A1 and A2) reveal that spherical oxides mainly consists of O, Cr and Ti. In addition, the content of Ti (higher than 20 at. %) is higher than that of Cr (less than 17 at. %). Combining the results of XRD presented in Figure 5a and EDS results, the spherical oxide is mainly composed of TiO_2_. It should be indicated that loose and porous TiO_2_ oxide has poor resistance to protect the matrix against oxidation.

It is shown in Figure 6d that oxide particles are finely and evenly distributed on the surface of RTM specimen. It is observed that the surface of the sample is unevenly distributed with pyramidal oxide and prismatic oxide, and the sizes of oxides range from 0.2 to 0.5 μm. Combining the results of XRD presented in Figure 5c and the EDS analysis in Figure 6f (Point B1, B2), it is inferred that pyramidal oxide and prismatic oxide are mainly composed of Cr_2_O_3_.

Figure 6h shows that the surface of LTB specimen is continuously and densely covered with oxidation product, which is beneficial for the dispersion of the internal stress in the oxidation layer and prevents further oxidation [34]. Moreover, it is observed that the oxidation product is likely to form three-dimensional triangular and small-sized spherical shapes and the sizes range from 0.1 to 0.5 μm. On the basis of EDS analysis in Figure 6i (Point C1and C2) and XRD pattern in Figure 5e, it is concluded that the main component of three-dimensional triangular oxide and small-sized spherical oxide are Cr_2_O_3_. 

Combining the results of XRD presented in Figure 5 and the EDS analysis in Figure 6 (zone A, zone B and zone C), the surface of SHT, RTM, and LTB specimens are mainly composed of Cr_2_O_3_ and TiO_2_. Furthermore, the Ti content in the surface of LTB specimen is lowest compared to that of RTM and SHT specimens. It can be inferred that the outward diffusion of Ti is inhibited by LTB processing.

### 3.5. Cross-Section Microstructure and Composition Distribution of Oxide Scales

**Figure 7:**
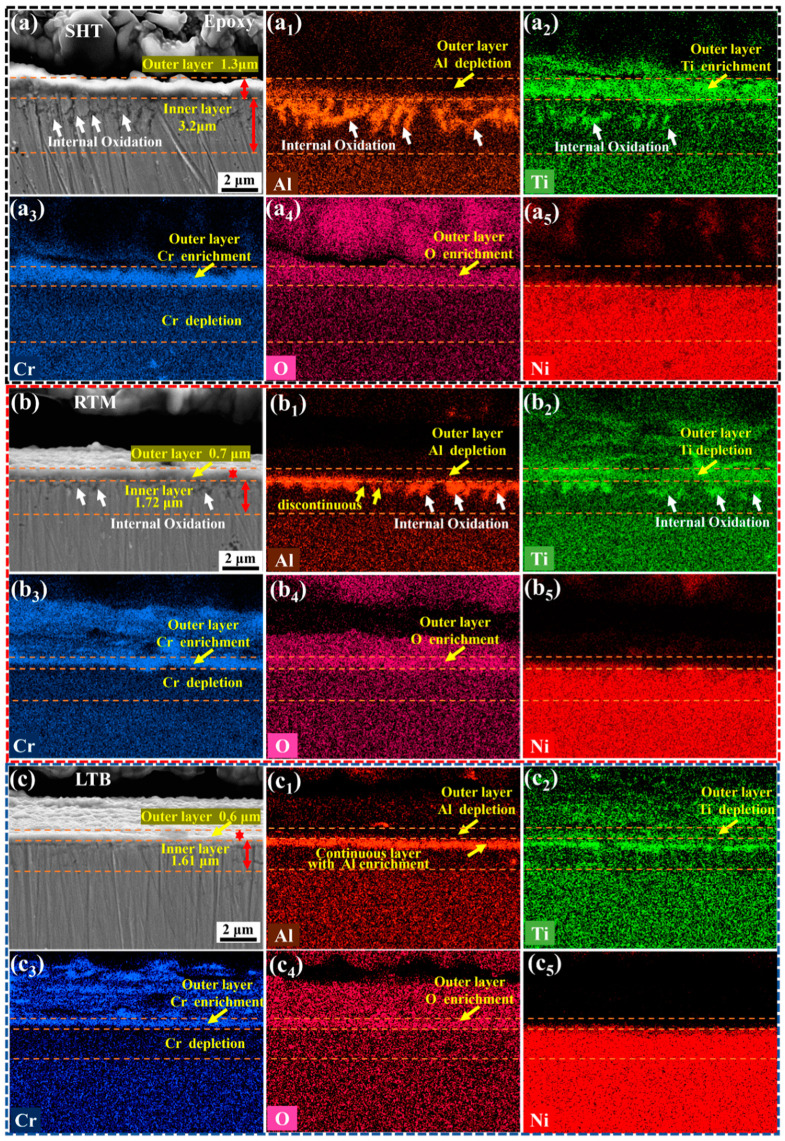
The cross-sectional images and EDS elemental mappings for different specimens after oxidation at 700 °C for 100 h: (**a**) SHT specimen; (**a_1_**–**a_5_**) Al, Ti, Cr, O and Ni mapping in the cross-section of SHT specimen, respectively; (**b**) RTM specimen; (**b_1_**–**b_5_**) Al, Ti, Cr, O and Ni mapping in the cross-section of RTM specimen, respectively; and (**c**) LTB specimen; (**c_1_**–**c_5_**) Al, Ti, Cr, O and Ni mapping in the cross-section of LTB specimen, respectively.

In order to further investigate the oxidation mechanism of GH738 superalloy, it is important to analyze the cross-section morphology of the oxide layer. Figure 7 shows the cross-section morphology and EDS elemental mappings of SHT, RTM, and LTB specimens after oxidation at 700 °C for 100 h. It can be seen that the oxide scales of specimens consist of two layers: a continuous outer oxide layer and an inner oxide layer.

Figure 7a shows the cross-section morphology and EDS elemental mappings of the SHT specimen. The thicknesses of the outer and inner oxide layers are approximately 1.3 μm and 3.2 μm, respectively. EDS elemental mapping in Figure 7a_1_–a_4_ shows that Cr, O, and Ti are rich in the outer oxide layer to form Cr_2_O_3_ and TiO_2_, while a small amount of Al_2_O_3_ forms beneath the outer oxide layer. It can be known from Ellingham diagram that Cr oxidizes at higher oxygen partial pressure than Al. As a result of this, Cr_2_O_3_ forms at the outer layer and Al_2_O_3_ forms beneath the outer layer [51]. Furthermore, the inner oxide layer shows severe internal oxidation (rich-Al,Ti oxides) (labeled by the white arrow) which occurs along the grain boundaries with a depth of about 3 μm. However, the depth of internal oxidation in RTM specimen is about 1μm, lower than that of the SHT specimen (Figure 7b).

Figure 7b shows the cross-section morphology and EDS elemental mappings of the RTM specimen. The thickness of the outer oxide layer is about 0.7 μm, while that of the inner oxide layer is about 1.72 μm. Figure 7b_1_–b_4_ show that the outer oxide layer is mainly rich in Cr and O to form Cr_2_O_3_. Al is more continuously distributed at the matrix/outer oxide layer interface of RTM specimen than that of SHT specimen. The internal oxidation of RTM specimen is much lower than that of SHT specimen.

Figure 7c shows the cross-section morphology and EDS elemental mappings of LTB specimen. The outer oxide layer of LTB specimen is with a thickness of approximately 0.6 μm and it is enriched with Cr, O, and a small amount of Ti. Combined with XRD pattern in Figure 5 and EDS results in Figure 6i, it is found that lots of Cr_2_O_3_ and minor amount of TiO_2_ are formed on the surface of LTB specimen. Furthermore, Figure 7c_1_ shows that a dense and continuous Al_2_O_3_ layer has formed at the interface between matrix and the outer oxide layer. Therefore, no obvious internal oxidation is found. Moreover, the continuous protective Al_2_O_3_ layer is strongly adhered to the superalloy and suppresses the outward diffusion of Ti and inward diffusion of O [6], which significantly improves the oxidation resistance of LTB specimen.

### 3.6. High Temperature Oxidation Mechanism of GH738 Superalloy

**Figure 8:**
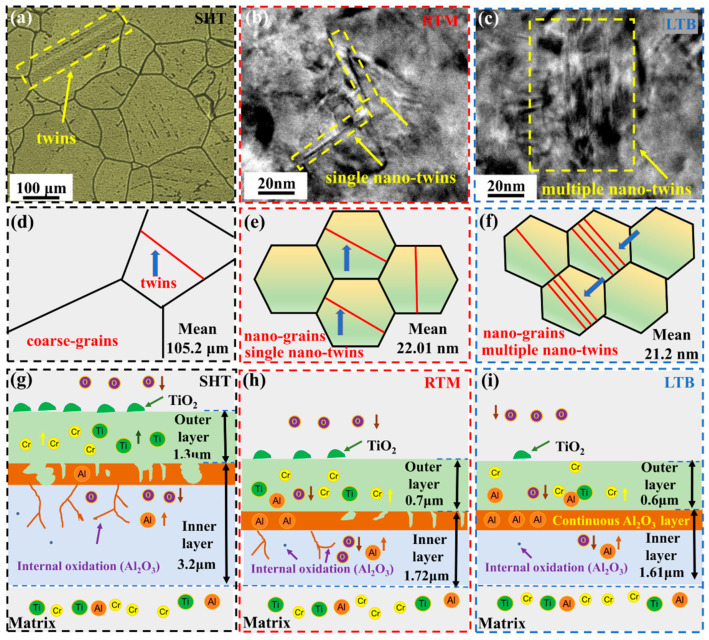
(**a**–**c**) The twins in the specimens of SHT, RTM, and LTB, respectively; (**d**–**f**) the model of twins in the specimens of SHT, RTM, and LTB, respectively; (**g**–**i**) the cross-section model after oxidation at 700 °C for 100 h of SHT, RTM, and LTB specimens, respectively.

Figure 8 illustrates the oxidation mechanism of the GH738 superalloy. It is found that low temperature burnished GH738 superalloy produces a continuous and dense Al_2_O_3_ layer at the interface between outer oxide layer and matrix, which improves the high temperature oxidation resistance.

Figure 8a is the OM image of the SHT specimen. Furthermore, Figure 8b,c are the TEM images of the RTM and LTB specimens, respectively. Figure 8d–f shows the model of microstructure of the SHT, RTM, and LTB specimens, respectively. As is shown in Figure 8a–c, the SHT and RTM specimens form single twins while the LTB specimen forms single twins and multiple-fold twins. It is observed that nano-grains have formed on the surface layer of the RTM and LTB specimens, while the surface layer of SHT specimen is composed of coarse grains. Figure 8g–i present the cross-sectional model of the SHT, RTM, and LTB specimens, respectively, after oxidation at 700 °C for 100 h. According to the analysis of Figure 6 and Figure 7, the schematic model (Figure 8g–i) is drawn.

According to the previous results (Figure 4), the order of high temperature oxidation resistance is LTB specimen > RTM specimen > SHT specimen. The possible reasons can be described as follows.

During high temperature oxidation of alloys, the relative diffusion rate of metal elements depends on several factors, including the Gibbs free energy of formation of the oxide, kinetics, and microstructure (grain size and crystal defects) [57,58]. Although, according to the thermodynamics data, the Gibbs free energy of the formation of Al_2_O_3_ (ΔG^Θ^ = −935.12 kJ/mol) is the most negative, comparing with that of TiO_2_ (ΔG^Θ^ = −764.35 kJ/mol) and Cr_2_O_3_ (ΔG^Θ^ = −575.45 kJ/mol) [59], a continuous Al_2_O_3_ layer could not form due to the content of Al being lower than the critical concentration required to form a continuous Al_2_O_3_ layer [60] in the SHT specimen. The Cr content (19.31 wt. %) is much higher than that of Ti (3.13 wt. %) in the GH738 superalloy. Therefore, from the viewpoint of kinetics, the growth rate of Cr_2_O_3_ is higher than that of TiO_2_, which results in the fast formation of Cr_2_O_3_ in the outer oxide layer. In the meantime, TiO_2_ grows not only in the outer oxide layer but also at the outer oxide layer/air interface due to the outward diffusion of Ti through Cr_2_O_3_ [59,61] (Figure 8g). The island TiO_2_ forms a loose oxide layer on the top surface of outer oxide layer (Figure 8g). It can be inferred that the loose outer oxide layer provides paths for the inward diffusion of O and makes severe internal oxidation [8].

After RTM processing, coarse grains (~105 μm) on the top surface of GH738 have been refined to nano-grains (~22 nm) and lots of nano-twins are produced, which makes the surface energy of the superalloy is high [34]. The nano-grained structure produces a high density of grain boundaries (GBs). The high density of GBs and nano-twins provide more diffusion paths for Cr, Al and Ti [62,63]. The Cr diffusion is faster along grain boundary than through matrix [51], which may result in the relatively compact and continuous Cr_2_O_3_ layer formed on the surface of RTM specimen. The outer oxide layer of RTM specimen is thinner and denser than that of SHT specimen (Figure 7a,b). It is speculated that the relatively compact and continuous Cr_2_O_3_ layer inhibits the inward diffusion of O and outward diffusion of Ti, which makes the content of TiO_2_ decrease in the outer oxide layer (Figure 6f and Figure 8h).

As shown in Figure 8g–i, in LTB specimen, a continuous Al_2_O_3_ layer is formed at the interface between the matrix and outer oxide layer, while the continuous Al_2_O_3_ layer does not form in the SHT and RTM specimens. This may be the main reason why the LTB specimen has the best high temperature oxidation resistance. Nano-grains and a large amount of single nano-twins and multiple nano-twins have been produced by LTB. Although, the average grain size of the LTB specimen is almost the same as that of RTM specimen, the twins density of LTB specimen is higher than that of RTM specimen for 127.73%. The higher twins density adds more diffusion paths for Cr, which may promote the formation of a more compact and continuous Cr_2_O_3_ layer on the surface of the LTB specimen (Figure 8i). In the meantime, the higher twins density promotes Al diffusion outward from the matrix and a continuous Al_2_O_3_ layer forms. The effective diffusion rates of O and metal elements through a continuous Al_2_O_3_ scale are relatively low [6,64], which improves the oxidation resistance of the LTB specimen. According to the above analysis, it is known that high temperature oxidation resistance of GH738 superalloy has been significantly improved by LTB processing.

## 4. Conclusions

The effects of RTM and LTB processing on surface microstructures and high temperature oxidation resistance of GH738 superalloys are investigated. The main conclusions can be summarized as follows:

(1) After oxidation for 100 h at 700 °C, the average mass gain of the SHT specimen is 0.3 (mg/cm^2^h), clearly higher than that of the RTM specimen for 45.45% and LTB specimen for 172.73%, which indicates that the LTB specimen has the best oxidation resistance. Surface treatment techniques (RTM, LTB) could not change the phase constitution of surface oxidation product, but they induce some differences in morphologies. The oxidation products on the surface of the SHT, RTM, and LTB specimens mainly consist of Cr_2_O_3_ and TiO_2_.

(2) Nanocrystalline surface layer is successfully obtained by RTM. The coarse grains on the top surface of RTM specimen have been refined to nano-grains (22.01 nm) which produces a higher density of grain boundaries than that of SHT specimen. Lots of single nano-twins also have formed on the top surface of RTM specimen. The twins density of RTM specimen (8.87 × 10^13^ number/m^2^) is higher than that of SHT specimen (3.81 × 10^9^ number/m^2^). The higher density of grain boundaries and twins density can provide more fast diffusion paths for Cr to form a dense and protective Cr_2_O_3_ layer whose average thickness is 0.7 μm, clearly thinner than that of SHT specimen. The dense and protective Cr_2_O_3_ layer inhibits Ti diffuse outward, O diffuse inward, and then makes the high temperature oxidation resistance of the RTM specimen better than that of the SHT specimen.

(3) Nano-grains (21.2 nm) and large amounts of single nano-twins and multiple nano-twins have been produced by LTB. Although the grain size of LTB specimen is almost the same as that of RTM specimen, the twins density of LTB specimen (2.02 × 10^14^ number/m^2^) is higher than that of RTM specimen (8.87 × 10^13^ number/m^2^) for 127.73%. The higher twins density provides more diffusion paths for Cr, which promote the formation of more compact continuous Cr_2_O_3_ layer. In the meantime, a continuous Al_2_O_3_ layer forms at the interface between matrix and out oxide layer in the LTB specimen. The continuous protective Cr_2_O_3_ and Al_2_O_3_ layer can further inhibit Ti diffusion outward and O diffusion inward, thus decreasing the oxidation rate of LTB specimen. Therefore, LTB significantly improves the high temperature oxidation resistance of GH738. The LTB specimen has the best high temperature oxidation resistance compared with SHT and RTM specimens. In the near future, nickel-based superalloy GH738 treated by LTB may be used for the blades and disks of industrial jet engines and gas turbines to improve their high temperature oxidation resistance in harsh environment.

## Figures and Tables

**Table 1:** Chemical composition of GH738 (wt.%).

C	Co	Al	Fe	Mo	B	Cr	Ti	Mg	Ni
0.04	13.25	1.46	0.2	4.38	0.006	19.31	3.13	0.006	Balance
